# Performance Evaluation of Gravity-Fed Water Treatment Systems in Rural Honduras: Verifying Robust Reduction of Turbidity and *Escherichia coli* during Wet and Dry Weather

**DOI:** 10.4269/ajtmh.17-0577

**Published:** 2018-08-08

**Authors:** Yolanda M. Brooks, Erika A. Tenorio-Moncada, Nisarg Gohil, Yuqi Yu, Mynor R. Estrada-Mendez, Geovany Bardales, Ruth E. Richardson

**Affiliations:** 1 School of Civil and Environmental Engineering, Cornell University, Ithaca, New York;; 2 Department of Environment and Development, Panamerican Agriculture University, Zamorano, Yeguare Valley, Municipality of San Antonio de Oriente, Francisco Morazán, Honduras

## Abstract

This is the first study to document the reduction of turbidity and *Escherichia coli* throughout the processes of full-scale gravity-fed drinking water plants (GFWTPs) and their downstream distribution systems in rural Honduras. The GFWTPs, which in these cases were designed by AguaClara, use standard treatment processes: coagulation, sedimentation, filtration, and chlorination. During the dry season, we measured *E. coli*, turbidity, and chlorine residual at five GFWTPs with < 1,000 connections and at three alternative piped-water systems in neighboring communities. Samples were evaluated from the raw water, settled water, filtered water, post-chlorination in the distribution tank, and at a distant-piped household connection. During the dry season, the treated water and household connections serviced by the GFWTPs met World Health Organization (WHO) recommendations for *E. coli* (< 1 most probable number [MPN]/100 mL). Alternative plants with the same water sources had comparable or higher *E. coli* and turbidity measurements posttreatment. We examined the performance robustness of two GFWTPs during the transition into the rainy season. The turbidity of the filtered water met WHO recommendations (< 1 nephelometric turbidity units). *Escherichia coli* was not detected in treated water, indicating that the two GFWTPs can consistently remove particulates and *E. coli* from source waters containing varying levels of turbidity. During two sampling events during the rainy season, *E. coli* was detected at the household connection of a GFWTP system with intermittent service and a substandard chlorine residual (geometric mean = 1.0 MPN/100 mL). Strategies to avoid contamination or inactivate *E. coli* in the distribution system are needed to ensure safe drinking water at the points of delivery, especially for systems with intermittent service.

## INTRODUCTION

Access to safe drinking water is essential for basic human health. Since 2000, the United Nation’s Millennium Development Goals and Sustainable Management Goals have spearheaded efforts to increase access of safe drinking water. In 2015, ∼75% of the global population, 4.2 billion people, had a piped water supply that was on premises.^1^ However, instances of fecal contamination in piped systems in low- and medium-income countries have been documented.^2–4^ Consumption of polluted water is a major contributor to the prevalence of diarrheal diseases.^5^

Treatment of water supplies is necessary to remove particulates, including microorganisms of public concern, in piped water supplies.^6,7^ In high-resource settings, water treatment is commonly accomplished through energy-intensive treatments. Processes typically present in these plants include in the order of operation: coagulation, flocculation, sedimentation, filtration, and disinfection. Energy-intensive treatment units are not feasible in low-resource settings where electricity is expensive, inconsistent, or simply not available. In theory and practice, gravity can replace electrical requirements for metering, mixing, and moving water through the drinking water treatment units. However, there is little research evaluating the performance of centralized gravity-powered water treatment systems to remove particulates and microorganisms (e.g., fecal indicators such as *Escherichia coli*) within the treatment units.

In 2010, 86% of the Honduran population had piped water on premises.^8^ Piped supplies in Honduras have been shown to contain waterborne pathogens such as *Giardia* spp. and *Cryptosporidium* spp.^9^ There were an estimated 4.6 diarrheal incidences per child-year in Honduran children < 5 years old due in part to contaminated drinking water.^10^ In an observational cohort of hospital and clinic visits reported to the Honduran Secretary of Health during 2000–2004, there was an estimated 1:123 risk of diarrheal deaths in children < 5 years old.^11^

AguaClara is a collaboration between Cornell University, Ithaca, NY, and Agua Para el Pueblo, Tegucigalpa, Honduras, a Honduran nongovernmental organization (NGO). AguaClara designs centralized water treatment plants for small towns in rural Honduras. The plants include full-scale water treatment processes such as flocculation, floc blankets, plate settlers, stacked rapid sand filtration, and chlorination. The treatment processes and their downstream distribution systems are powered by gravity and provide treated water to household connections without relying on electricity. As of 2017, GFWTPs designed by AguaClara provide drinking water to 65,000 people in Honduras and India. Laboratory investigations of the treatment processes in the AguaClara plants have led to the invention of high-rate sedimentation tanks with floc blankets and ability to self-clean without moving parts^12^ as well as stacked rapid sand filters that can be backwashed without pumps.^13^

We are not aware of any studies that have investigated the reduction of turbidity and fecal indicator bacteria of GFWTPs during normal operation. Such investigations can provide a benchmark for improving the understanding of the capabilities and limitations of centralized, gravity-powered water treatment plants. In addition, documentation of the performance of GFWTPs will help accelerate adoption of these plants in small communities within high-income countries, which often struggle to afford highly mechanized treatment plants. In this study, we investigated microbial, chemical, and physical indicators of water quality along the treatment train and in household connections of five GFWTPs in rural Honduras. The first objective of our study was to evaluate and compare the reduction of culturable *E. coli* and turbidity, and concentrations of chlorine residual (storage tank and distribution system only) along the treatment trains during dry weather of five GFWTP systems and three nearby drinking water systems—two with chlorination-only and one multistage filtration plant. The second objective was to evaluate the concentrations of culturable *E. coli*, turbidity, and free chlorine in the treatment processes and delivered water of two of the GFWTPs during the transition from the dry season to the rainy season, when the water quality of the source water can degrade drastically.

## METHODS

### Site descriptions.

**Figure 1.**
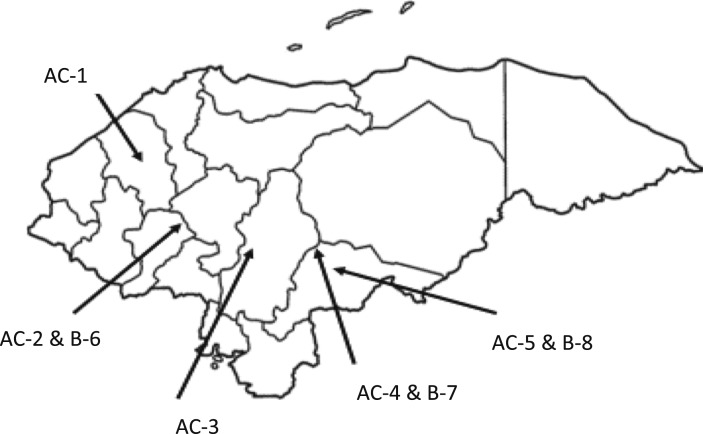
Map of the locations of the gravity-fed drinking water treatment plants (AC-1 through 5) and the alternative treatment systems (B-6 through 8) that were evaluated in this study. Table 1 contains a description of each plant.

During January 2016, we visited five GFWTPs designed by AguaClara in five communities in southern and western Honduras (labeled AC-1 through 5; Figure 1). Their construction was overseen by a Honduran NGO, Agua Para el Pueblo. Honduras has regulatory standards regarding the quality of treated water.^14^ The water supplies of all of the plants are surface waters such as springs or rivers that are delivered to the plants via gravity conduction lines. The Swiss Development Cooperation funded the construction of all of the plants except AC-3, which was funded via private donations. Information regarding the population serviced, year built, and production volumes of all plants are listed in Figure 1. AC-1 through 5 had the following processes: grit removal, flocculation, floc blankets, lamellar sedimentation, stacked rapid sand filtration (except AC-3), and chlorination and storage in a distribution tank before entering the distribution system that delivered the treated water to household premises (Table 1 and Supplemental Information S1). The GFWTPs are operated, maintained, and governed by a water board comprising elected citizens within the distribution system and do not have Water Safety Plans modeled after World Health Organization (WHO) recommendations. At each GFWTP, turbidity measurements are routinely taken at least two times per day in the raw water, settled water, and, where applicable, filtered water. Each treatment plant records the calculations of coagulant used and target chlorine dosage applied to the filtered water.

**Table 1:** Description of key treatment processes and features of each plant

Plant	Flocculation and sedimentation	Filtration	Chlorination	Population served	Production volume (L/s)	Year built	Location
AC-1	Y	Y	Y	6000	32	2014	San Nicolas, Santa Barbara
AC-2	Y	Y	Y	5000	20	2015	Jesus de Otoro, Intibuca
AC-3	Y	N	N*	2000	6	2009	Cuatro Comunidades, Francisco Morazan
AC-4	Y	Y	Y	4500	16	2016	Morocelí, El Paraíso
AC-5	Y	Y	Y	3800	14	2016	San Matías, El Paraíso
B-6	N	Y†	N*	1600	25	2000	Jesus de Otoro, Intibuca
B-7	N	N	Y	180	3	1982	Suyate, El Paraíso
B-8	N	N	Y	145	2	1995	San Marcos Abajo, El Paraíso

* Chlorination at AC-3 was inoperable on the sampling day.

† B-6 had three successive filtration steps, two gravel steps and a final rapid sand filter.

We visited drinking water treatment systems (B-6 through 8) with varying levels of treatment that had the same raw water supply as one of the GFWTPs (Figure 1). These were a multistage filtration plant (B-6) and two chlorination-only plants (B-7 and B-8). We were unable to determine the funding mechanism for the construction of B-7 and B-8. Construction of B-6 was funded by the Swiss Development Cooperation. B-6 has three successive filtration steps (two gravel steps using dynamic and roughing filtration followed by a slow sand filter) and equipment for chlorine gas disinfection. B-6 was not chlorinating the finished water during our visit. The alternative treatment plants are governed by a local board and do not have Water Safety Plans modeled after WHO recommendations.

### Description of sampling events and sampling points.

In base flow conditions during January 2016, we visited five GFWTPs (AC-1 through 5) and three alternative treatment plants (B-6 through 8). At each GFWTP, we sampled from five points within the AC GFWTPs and their downstream distribution systems: raw water after grit removal, settled water, filtered water (except AC-3), distribution tank, and at one of the furthest household connections from the distribution tank. At B-6, we collected water samples from the raw water and from each of the three successive filtration steps. At the chlorination-only treatments (B-7 and B-8), we collected samples from the raw water and at one of the furthest household connections from the distribution tank. For samples from the tap, water was run for at least 5 minutes before the sample was taken. Within each sampling point, we collected duplicate samples of water.

During May–July 2016, we visited the two GFWTPs, AC-4 and AC-5, during six sampling events: three sampling events occurred in the dry season during May 2016 (dry weather) and three sampling events occurred during the onset of the rainy season during June–July 2016 (wet weather) < 24 hours after a rain event that occurred at or in the vicinity of the sources of the raw water. At each GFWTP, we took samples from five sampling points previously listed in the GFWTPs. During the May–July 2016 sampling campaign, water was collected from the same household connection in each town. Within each sampling point, water samples were collected in triplicate.

### Measurements of physical and chemical qualities and concentrations of *E. coli* in water samples.

During the January 2016 and May–July 2016 sampling campaigns, for chemical parameters we collected 50 mL from the sampling points listed previously. During January 2016, we measured turbidity using a MicroTPI Field Portable Turbidimeter (HF Scientific, Fort Myers, FL) with a detection limit of 0.01 NTU. During January 2016, the concentrations of free chlorine (mg/L-Cl_2_) from the distribution tank and at the household connection were determined using a free chlorine color disc test (Hach, Inc., Loveland, CO). During May–July 2016, turbidity of the treatment processes contained in the water treatment plant (raw water after grit removal, sedimented water, and filtered water) were measured by the plant operator with a MicroTPI Field Portable Turbidimeter (HF Scientific), whereas free chlorine and turbidity at the distribution tank and at the household connection were measured with a digital colorimeter DR-890 (Hach, Inc.; detection limits: 0.01 mg/L and 1 NTU, respectively). The lower limits of detection of the free chlorine measurements during the January 2016 were 0.02 mg/L. Values below the detection limits were reported as less than the detection limit and evaluated at one-half of the detection limit in subsequent analyses and graphs. During the May–July 2016 sampling event, the turbidity measurements of the raw water were measured by the plant operators within 24 hours of the sampling event during dry weather in AC-4 and AC-5. Recent (within 30 days) turbidity measurements are available online for the raw water, sedimented water, and filtered water of the GFWTPs at http://aguaclara.github.io/settings.html).

Replicate 100-mL samples (duplicates in January 2016 and triplicates in May–July 2016) were analyzed for concentrations of *E. coli* in water samples using the manufacturers’ instructions of the compartment bag test (CBT; AguaGenX, LLC, Chapel Hill, NC).^15^ Specifically, samples of water were aseptically collected using 100-mL Whirl-Pak^®^ Thio Bags^®^ (Nasco, Fort Atkinson, WI) and stored in a cooler on ice for ≤ 6 hours. During the January 2016 sampling campaign, the CBT assays were incubated at ambient temperatures, 25–30°C for 40–48 hours (time varied based on incubation temperatures as per the manufacturer’s instructions), whereas the CBT assays performed during the May–July 2016 sampling campaign were incubated at 35°C for 24 hours.^16,17^ The quantification range of each CBT assay is 1–100 MPN *E. coli*/100 mL. During the May–July 2016 sampling campaign, one replicate of the raw water was diluted 10× with bottled water and another was diluted 100× to ensure at least one replicate was within the quantification range. Negative controls were bottled water and were processed every day of water collection. All negative controls were negative for the presence of *E. coli*. For all samples, we reported the geometric mean concentrations of *E. coli* calculated from the replicates of each sample. During the January 2016 and the May–July 2016 sampling campaigns, there were distinct lower limits of detection of the concentrations of *E. coli* in a water sample: 0.5 and 0.3 MPN/100 mL, respectively, because of the distinct number of replicates per sample (two versus three, respectively). Assays that did not have detectable *E. coli* were reported as less than the lower detection limit and evaluated at one-half of the detection limit in subsequent analyses and graphs. Replicates of each assay that were above the upper limit of detection, 100 MPN/100 mL, were reported as > 100 MPN/100 mL in subsequent analyses and graphs.

### Statistical analyses.

All analyses were performed in IBM SPSS Statistics for Windows, Version 23.0 (IBM, Corp., Armonk, NY) and significance was considered at α = 0.05. Within the data gathered from the evaluation of the transition of the rainy to dry season over six sampling events in AC-4 and AC-5, Mann–Whitney tests analyzed the differences between the concentrations of *E. coli* and turbidity measured at the raw water during the dry and wet weather sampling events.

## RESULTS

### Turbidity reduction in gravity-fed water treatment systems and alternative systems.

**Figure 2.**
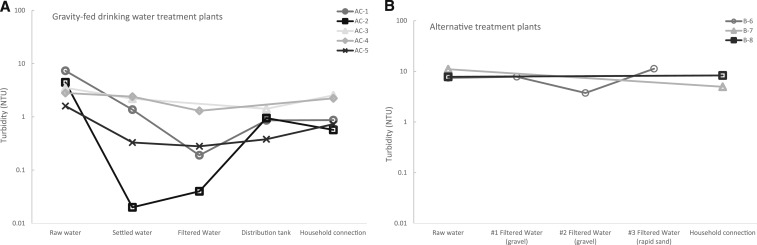
Comparison of turbidity measurements (NTU) from the treatment train processes and at a distant household connection from (**A**) from five gravity-fed water treatment plants (AC-1 through 5)^a^; and (**B**) from three alternative treatments (B6, B-7, and B-8 )^b,c^ during the dry season. Each data point represents one measurement. Display of the data using an arithmetic scale is displayed in Supporting Information Figure S2. ^a^AC-3 did not have a filtration process. ^b^Chlorination was the only treatment for B-7 and B-8. ^c^B-6 included three consecutive filtration processes. Chlorination was not functioning during sampling and we did not collect a sample at a household connection or in the distribution tank.

Turbidity was measured at five sampling locations within each of the GFWTPS (only four sampling locations in AC-3) in the raw water, settled water, filtered water, in the distribution tank, and at one of the furthest household connections. All GFWTPs showed a decrease in turbidity from raw water to treated water, although the extent of the reduction varied. At AC-1, AC-2, AC-4, and AC-5, the turbidity decreased from the raw water (1.6, 2.8, 7.4, and 4.5 NTU, respectively) to the filtered water (0.3, 1.3, 0.2, and 0.02 NTU, respectively). Turbidity levels at a distant household connection were 0.7, 2.2, 0.9, and 0.6 NTU, respectively (Figure 2A). There was no filtration process at AC-3, and the turbidity decreased from the raw water (3.5 NTU) to the settled water (2.2 NTU), followed by a slight increase measured at the household connection (2.5 NTU; Figure 2A).

Turbidity was also measured in the alternative treatment systems. In B-7 and B-8, the piped water systems only included chlorination and were sampled in the raw water and at one of the furthest household connections. In B-8, there was a slight increase in the turbidity measured from the raw water (7.8 NTU) to household connection (8.3 NTU; Figure 2B). In B-7, the turbidity decreased from the raw water (11 NTU) to the household connection (5.0 NTU; Figure 2B). B-6 was a multistage filtration system where turbidity was measured at the raw water and after each of the three filtration steps. At B-6, the turbidity oscillated between the raw water and after the three consecutive filtration steps (3.7–11.3 NTU; Figure 2B).

In addition, the turbidity measured at the household connection of AC-5 were > 10-fold less (0.7 NTU) than the household connection of the corresponding alternative treatment plant (B-8; 8.3 NTU). Comparing the turbidity of the household connection in AC-4 and B-7, AC-4 had less turbid water (2.2 versus 5.0 NTU).

### Free chlorine measurements in the distribution tank and at the household connections of GFWTPs and alternative systems.

Free chlorine was measured in the distribution tank and at one of the furthest household connections of the five gravity-fed water treatment systems. Free chlorine was also measured at one of the furthest household connection of the two chlorination-only systems, B-7 and B-8. All GFWTPs except AC-3 had detectable residual chlorine in the distribution tanks and at the household connections (Table 2). In AC-2, residual chlorine increased from the distribution tank to the household connection (Table 2). Chlorinaton was offline on the sampling date of AC-3. Because a single parcel of water was not followed from the plant to the tap, it is possible that variability in dosing caused slight differences in chlorine between the distribution tank and household tap. The free residual chlorine at the household connections of the alternative systems (B-7 and B-8) are listed in Table 2 and were comparable with the range of concentrations at the household connections of the GFWTP systems.

**Table 2:** Free residual chlorine (mg/L) measured in the stored and chlorinated water and at a distant household connection of five gravity-fed water treatment plants (AC-1 through AC-5) and three alternative treatment plants (B-6 through B-8) during dry weather in January 2016

	Mean free residual chlorine concentration, mg/L							
	Gravity-fed water treatment plants					Alternative treatment plants		
	AC-1	AC-2	AC-3*	AC-4	AC-5	B-6*	B-7	B-8
Distribution tank	**0.9**	0.14	0.01†	0.27	0.2	ND‡	ND‡	ND‡
Household connection	**1.3**	**0.4**	0.01†	**0.25**	0.1	ND‡	**0.23**	0.05

Measurements within the World Health Organization recommendations (0.5–2 mg/L free chlorine in the distribution tank and 0.2–2 mg/L at the household) are represented in bold.

* Chlorination was offline at AC-3 and B-6 during the sampling event.

† The lower detection limit was 0.02 mg/L. Measurements below the detection limit were reported at one-half of the lower detection limit.

‡ ND = no data collected. We were unable to collect samples from the distribution tank in B-6, B-7, and B-8, and at a distant household connection for B-6.

### Reduction of *E. coli* throughout the treatment processes and distribution systems of the gravity-fed water treatment systems and alternative treatment systems.

**Figure 3.**
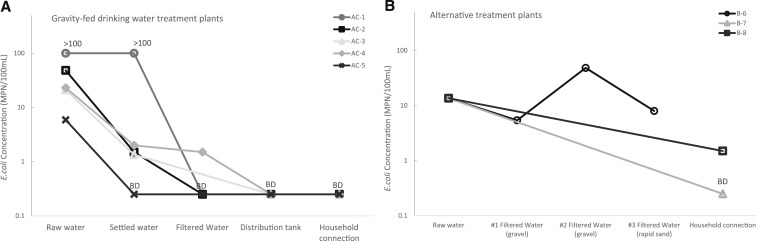
Comparison of the geometric mean concentrations of *E. coli* (MPN/100 ml) from the treatment train processes and at a distant household connection (**A**) from five gravity-fed water treatment plants (AC-1 through 5)^a^; and (**B**) from three alternative treatments (B6, B-7, and B-8)^b,c^ during the dry season. Data points that are outside the quantification range (0.5-100 MPN/100 ml) are represented at one half the lower detection limit with the label BD (below detection) or at the upper detection limit. Each data point represents the geometric mean of two replicates. ^a^AC-3 did not have a filtration process. ^b^Chlorination was the only treatment for B-7 and B-8. ^c^B-6 included three consecutive filtration processes. Chlorination was not functioning during sampling and we did not collect a sample at a household connection.

In both the alternative treatment systems and the GFWTP systems, concentrations of *E. coli* were measured in the same sampling points as the turbidity measurements. At each sampling point, the concentrations of *E. coli* were measured in duplicate and the geometric mean was reported. In all AC GFWTPs, there was an overall reduction of the mean concentrations of *E. coli* from raw water to the distribution tank (Figure 3A). At least a 10-fold decrease of the geometric mean concentrations of *E. coli* between raw water and settled water were measured in AC-2 through 5, where the concentrations of *E. coli* in the raw and the settled water were both above the upper detection limit for CBT (> 100 MPN/100 mL; Figure 3A). There was detectable *E. coli* (≥ 0.5 MPN/100 mL) in the filtered water before chlorination in AC-4. In all GFWTPs, there was no detectable *E. coli* (< 0.5 MPN/100 mL) in the distribution tank and at the household connections serviced, indicating that AC1 through 5 had the following reduction efficiencies: > 99.5, > 99.0, > 97.6, > 97.8, and > 91.5%, respectively.

The geometric mean concentrations of *E. coli* measured in the raw water of B-6 was 13.6 MPN/100 mL and oscillated with successive filtration steps (between 5.4 and 48.3 MPN/100 mL) with water leaving the final filtration step with 8.0 MPN/100 mL (Figure 3B), representing 41.2% overall reduction. *Escherichia coli* was detected at the household connection of B-8 (geometric mean = 1.5 MPN/100 mL) representing 89.0% reduction, whereas *E. coli* was not detected at the household connection in B-7 (> 96.3% reduction of *E. coli*).

### Transition to the rainy season: reduction of turbidity throughout the processes and distribution systems of two GFWTPs.

**Figure 4.**
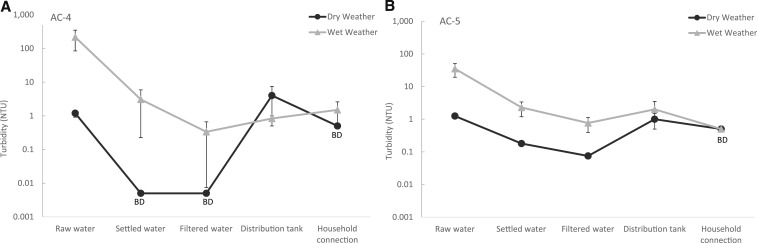
The mean turbidity measured in the treatment processes and downstream distribution systems of two gravity-fed water treatment plants, AC-4 (**A**) and AC-5 (**B**) during three dry weather and three wet weather sampling events between May–July 2016. Data points where all measurements were below the lower detection limit (0.01 NTU for raw water, settled water and filtered water; and 1 NTU for distribution plant and at the household connection) were represented at one-half of the detection limit with the title BD (below detection). The error bars represent one standard error.

During six sampling events spanning May–July 2016, turbidity was measured at five sampling points in AC-4 and AC-5: raw water, settled water, filtered water, in the distribution tank, and at one of the furthest household connections. In AC-4 and AC-5, the mean turbidities of the raw water in wet weather (and dry weather) were 77.3 (1.7) and 45.3 NTU (1.3 NTU), respectively (Figure 4A–B). The turbidities of the raw water during wet weather in AC-4 and AC-5 were not significantly larger than during dry weather (*P* = 0.10). In both plants, the mean turbidity consistently decreased from the raw water to the household connections during dry and wet weather conditions (Figure 4A–B).

### Transition to the rainy season: free residual chlorine measurements in the distribution tank and at the household connections of two gravity-fed water systems.

During six sampling events spanning May–July 2016, free chlorine was measured in the distribution tank and at one of the furthest household connections of the AC-4 and AC-5 systems. The mean target dosages of chlorine applied to filtered water were calculated by the plant operators and their ranges are listed in Table 3. Independent of weather conditions, the mean concentrations of free chlorine in the distribution tank and at the household connection were larger in AC-4 than in AC-5. During dry and wet weather in both plants, the mean concentrations of free chlorine in the distribution tank were larger than at the household connection (Table 3).

**Table 3:** The range of target chlorine concentrations and mean concentrations of free chlorine (mg/L) measured in the distribution tank and at a household connection of two gravity-fed drinking water treatment systems (AC-4 and AC-5) sampled in wet and dry weather during May–July 2016

	Mean free residual chlorine concentration, mg/L ± std. error			
	AC-4		AC-5	
	Dry weather	Dry weather	Dry weather	Wet weather
Target chlorine concentration (range measured across all sampling dates)	1.01–1.04		0.7–1.0	
Distribution tank	0.43 ± 0.08	0.51 ± 0.08	0.23 ± 0.15	0.51 ± 0.08
Household connection	0.36 ± 0.12	0.27 ± 0.09	0.02 ± 0.02	0.27 ± 0.09

The lower detection limit was 0.02 mg/L.

### Transition to the rainy season: reduction of *E. coli* throughout the treatment processes and in the distribution systems of two gravity-fed water treatment systems.

**Figure 5.**
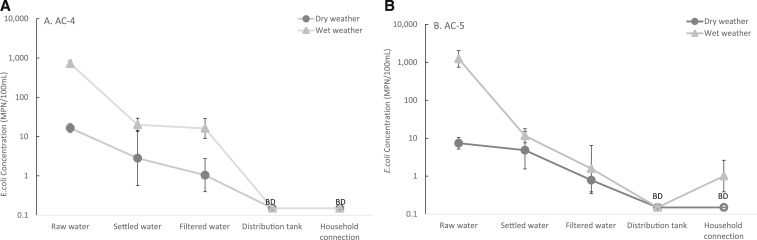
Geometric mean concentrations of *E.coli* in the treatment processes and downstream distribution systems of two gravity-fed water treatment plants, AC-4 (**A**) and AC-5 (**B**) during three dry weather and three wet weather sampling events between May–July 2016. Data points where all measurements were below the lower detection limit, 0.3 MPN/100 mL, were represented at one-half of the detection limit with the title “BD” (below detection). Each data point represents the geometric mean of three sampling dates. The error bars represent one standard error.

During each of the six sampling events, concentrations of *E. coli* were measured in each of the sampling points alongside turbidity. The geometric mean concentrations of *E. coli* in the raw water of AC-4 (and AC-5) during dry and wet weather were 16.5 and 718 MPN/100 mL, respectively (7.4 and 1,231 MPN/100 mL, respectively; Figure 5A–B). The concentrations of *E. coli* in the raw water in AC-4 and AC-5 were not significantly larger during wet weather than dry weather (*P* = 0.10). During wet weather, the largest decrease of the geometric mean concentrations of *E. coli* in the plants in AC-4 and AC-5 occurred between raw water and settled water (718 to 20.0 MPN/100 mL and 1,231 to 11.6 MPN/100 mL, respectively; Figure 5A–B). After subsequent treatment processes (sedimentation and filtration) in AC-4 and AC-5, there were similar concentrations of *E. coli* during wet and dry weather. In wet weather in AC-4 and AC-5, reduction efficiencies between raw water and the distribution tank were consistently > 96%. During dry and wet weather in AC-4 (and dry weather in AC-5), *E. coli* was not detected (< 0.3 MPN/100 mL) at either the distribution tank or the household connection (Figure 5A–B). However, *E. coli* was detected at the household connection during two wet weather sampling events in AC-5 and had a geometric mean concentration of 1.0 MPN/100 mL across the three wet weather sampling events.

## DISCUSSION

To our knowledge, this is the first peer-reviewed study to evaluate the reduction of *E. coli* and turbidity during the treatment trains of GFWTPs. During dry weather in January 2016, we visited eight treatment plants, five GFWTPs (AC-1 through 5), and three alternative plants (B-6 through 8), in rural Honduras. Within the GFWTPs, there was an overall reduction of turbidity from the raw water to the household connection. Reduction throughout the treatment train was dependent on the plant, with AC-3 and A-4 having the smallest reduction in turbidity from raw water to the household connection (Figure 2A). The absence of a filtration unit could explain the lower reduction efficiency in AC-3. In AC-4, the muted reduction of turbidity may be due to insufficient coagulant added to the flocculation tanks or a decreased residence time in the sedimentation tanks. Compared with their companion GFWTP systems, the alternative systems had higher measurements and lower free residual chlorine at the points of delivery (except B-7 which had comparable chlorine levels with AC-4 near 0.2 mg/L). Also, *E. coli* was detected at a point of delivery in B-8. The results from a singular sampling event during dry weather indicated that the drinking water at one of the furthest household connections from the gravity-fed treatment systems was of higher quality than the evaluated alternative systems. Because of variables such as the age of treatment plant and its downstream distribution system, hydraulic conditions, variation in water quality conditions, and size of the distribution systems, we are unable to conclude that improved water quality of the gravity-fed water treatment systems was due solely to the design of the GFWTP systems.

We also evaluated the performance of two GFWTPs, AC4 and AC-5, during a multi-week sampling campaign during the transition from dry to wet seasons during May–July 2016. The wet weather sampling took place during the first major rain event of the 2016 rainy season when overland storm runoff washes ground-deposited feces and particles into the surface water supplies (“first-flush” effect). During wet weather, the ranges of turbidity in the raw water of AC-4 and AC-5 were 10.04–271.40 NTU and 0.75–314.70 NTU, respectively. Although turbidity and *E. coli* levels increased in the source waters after the onset of the rainy season (Figures 4A–B and 5A–B), the water in the distribution tanks consistently met the WHO recommendations for *E. coli* (< 1 MPN/100 mL; Table 4), indicating that the GFWTPs robustly removed fecal pollution in the raw water. The microbial quality of the water was consistently maintained at the point of delivery in AC-4 (Table 4, Figure 5A). Despite the high quality of produced water even during the rainy season, *E. coli* was detected at a household connection in AC-5 during two sampling events (geometric mean concentrations of 2.1 and 3.2 MPN/100 mL during each sampling event; Table 4, Figure 5B). The contamination found during two sampling events suggest that at the time of sampling, there were fecal intrusion(s) in the distribution system and insufficient residual chlorine to inactivate microorganisms of public concern in the water of the distribution system.

**Table 4:** Comparison of the performance of two gravity-fed drinking water treatment systems (AC-4 and AC-5) during wet and dry weather to the WHO recommendations for turbidity, *Escherichia coli*, and residual chlorine of treated drinking water

Parameter		Mean in the gravity-fed water treatment systems accross six sampling dates (range)		WHO recommendations
		AC-4	AC-5	
Turbidity (NTU)	Before disinfection	1.17 (< 0.01–6.00)	0.5 (< 0.01–3.00)	< 1 with full treatment*
	Distribution tank	2.17 (< 0.01–11.00)	1.17 (< 0.01–5.00)	NA†
	At the household connection	0.7 (< 0.01–2)	< 0.01	NA†
*E. coli* concentrations (MPN/100 mL)	Distribution tank	< 0.3	< 0.3	< 1
	At the household connection	< 0.3	0.4‡ (< 0.3–3.12)	< 1
Residual chlorine (mg/L)	Distribution tank	0.47 (0.34–0.6)	0.29 (0.03–0.52)	0.5–5§
	At the household connection	0.30 (0.10–0.57)	0.08 (< 0.02–0.19)	0.2–2
Source		This study	This study	WHO (2010)

WHO = World Health Organization.

* Or < 5 NTU before disinfection in systems with limited or no treatment available.

† Not applicable.

‡ Geometric mean.

§ After 30 minutes of contact.

It is worth noting that the distribution system in AC-5 provided intermittent service during May–July 2016. Intermittent service may have contributed to *E. coli* contamination at the point of delivery during two wet weather sampling events. Comparably, a systemic review determined that improved water supplies had significantly larger loads of fecal contamination during rainy seasons compared with dry seasons.^18^ In addition, there were more occurrences of *E. coli* contamination and significantly larger concentrations of *E. coli* in distribution systems receiving intermittent supplies shortly after a rain event than during dry weather in Hubli and Dharwad, Karnataka, India.^19^ These results reinforce the need for routine monitoring of *E. coli* at points of delivery and increasing chlorine residual of intermittent water supplies to inactivate fecal intrusion within the distributions systems.

There are various pathways that could be responsible for the *E. coli* contamination observed in the distribution system in AC-5. A global review of intermittent water supplies determined that corrosion and depressurizing/repressurizing cycles put excess strain on the pipes and suggest that large volumes of intrusions may enter the pipes during extended periods of low pressure.^20^ In addition, backflush and low pressure were identified as mechanisms that introduced fecal contamination in distribution systems in Hubli and Dharwad Karnataka, India.^21^ A study of an intermittent water system in Arraiján, Panama, suggested that possible fecal intrusions were attenuated by longer supply durations, increased pressure in the distribution system, and a higher chlorine residiual.^22^ Therefore, increasing the service duration, water pressure, and/or chlorine residual in the distribution system of AC-5 could improve the quality of the intermittent water supply at the points of delivery and decrease associated health risks.

Overall, the largest reduction in turbidity and concentrations of *E. coli* in two GFWTPs during wet weather occurred between raw water and the settled water (Figures 4A–B and 5A–B), indicating that flocculation, floc blankets, and sedimentation are responsible for most of the reduction of particulate matter, including suspended microorganisms. Throughout the sampling events, the turbidity measured after filtration in AC-4 and AC-5 (except one wet weather sampling event in AC-4) were < 1 NTU, which met the WHO recommendations before disinfection.^23^ Compared with the large water system in Arraiján, Panama, servicing 263,000 people, overall the points of delivery of the two rural GFWTPs in Honduras had comparable turbidity and concentrations of residual chlorine (AC-4 only in dry weather).^22^

Our study indicates that the GFWTPs designed by AguaClara and built/operated by local labor produce high-quality drinking water and their treatment processes are robust against the increases in turbidity and fecal pollution in raw water that accompany rain events. However, intrusions in the distribution system can erode the accomplishments of the treatment processes. The data generated in our study provide a strong recommendation to initiate routine monitoring of the gravity-fed water treatment systems in rural Honduras. Monitoring schemes should include operational monitoring of *E. coli*, free residual chlorine, and turbidity throughout the treatment processes and the distribution system. Although turbidity and chlorine residual tests are already carried out locally at the GFWTPs, conventional efforts to monitor *E. coli* concentrations require high costs including transportation to a centralized laboratory and equipment and labor to perform the method. The data collected in our study demonstrated that routinely measuring *E. coli* is possible in low-resource settings using CBT and the data generated by the CBT agree with conventional *E. coli* enumeration methods such as Colilert and membrane filtration.^15,17,24^ Training plant operators or water board members to routinely monitor water safety will allow the identification of fecal intrusions, ensuring residual disinfection throughout the distribution system, and confirming the microbial and physicochemical quality of the water at the points of delivery. This training will also help move resource-limited communities closer to consistently meeting the UN’s Sustainable Management Goal 6.1 of “universal access to safe and affordable drinking water.”^1^

## Supplementary Material

Supplemental information
