# Supplementary material for: Performance Evaluation of Gravity-Fed Water Treatment Systems in Rural Honduras: Verifying Robust Reduction of Turbidity and Escherichia coli during Wet and Dry Weather
Source: Am J Trop Med Hyg. 2018 Aug 8;99(4):881–8. doi: 10.4269/ajtmh.17-0577 (PMC6159558; doi:10.4269/ajtmh.17-0577)
Supplement: Supplementary file 1 [file tpmd170577.SD1.pdf]

Supporting information for:

Title: Performance evaluation of gravity-fed water treatment systems in rural Honduras: verifying robust reduction of turbidity and *Escherichia coli* during wet and dry weather

Short title: Turbidity and *E.coli* decreases in gravity-fed water plants

Authors: Yolanda M. Brooks[a]; Erika A. Tenorio-Moncada[b]; Nisarg Gohil[a]; Yuqi Yu[a]**;** Mynor R. Estrada-Mendez[b]**;** Geovany Bardales[b]; Ruth E. Richardson[a]

[a]School of Civil and Environmental Engineering, Cornell University, Ithaca, NY

[b]Department of Environment and Development, Panamerican Agricultural University, Zamorano, Yeguare Valley, Municipality of San Antonio de Oriente, Francisco Morazan, Honduras, Central America

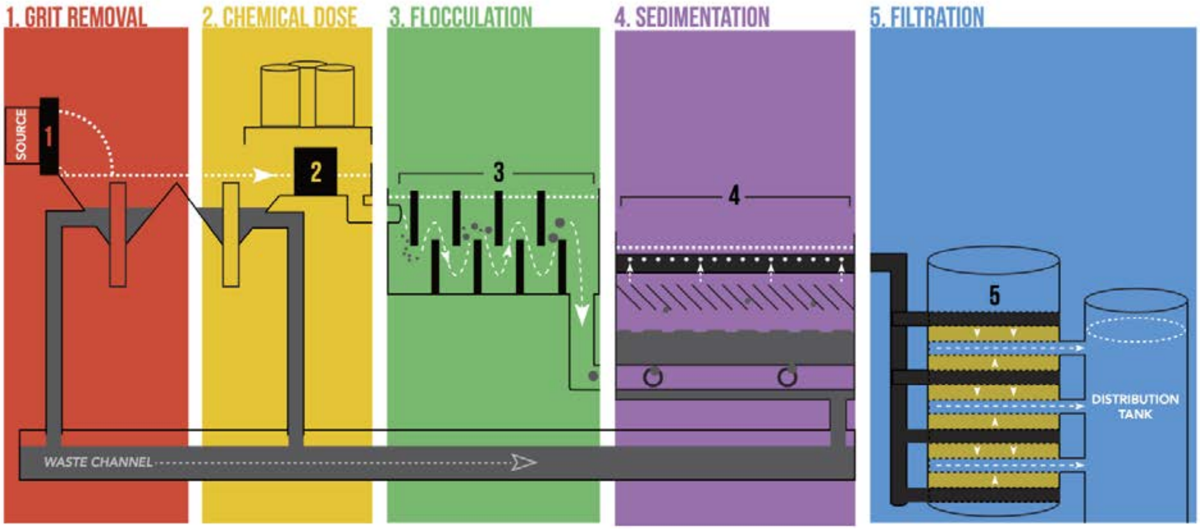

Figure S1: Treatment trains of full-scale, gravity-fed water treatment plants designed by AguaClara, a collaboration between Cornell University and a Honduran non-profit, Agua para el Pueblo.

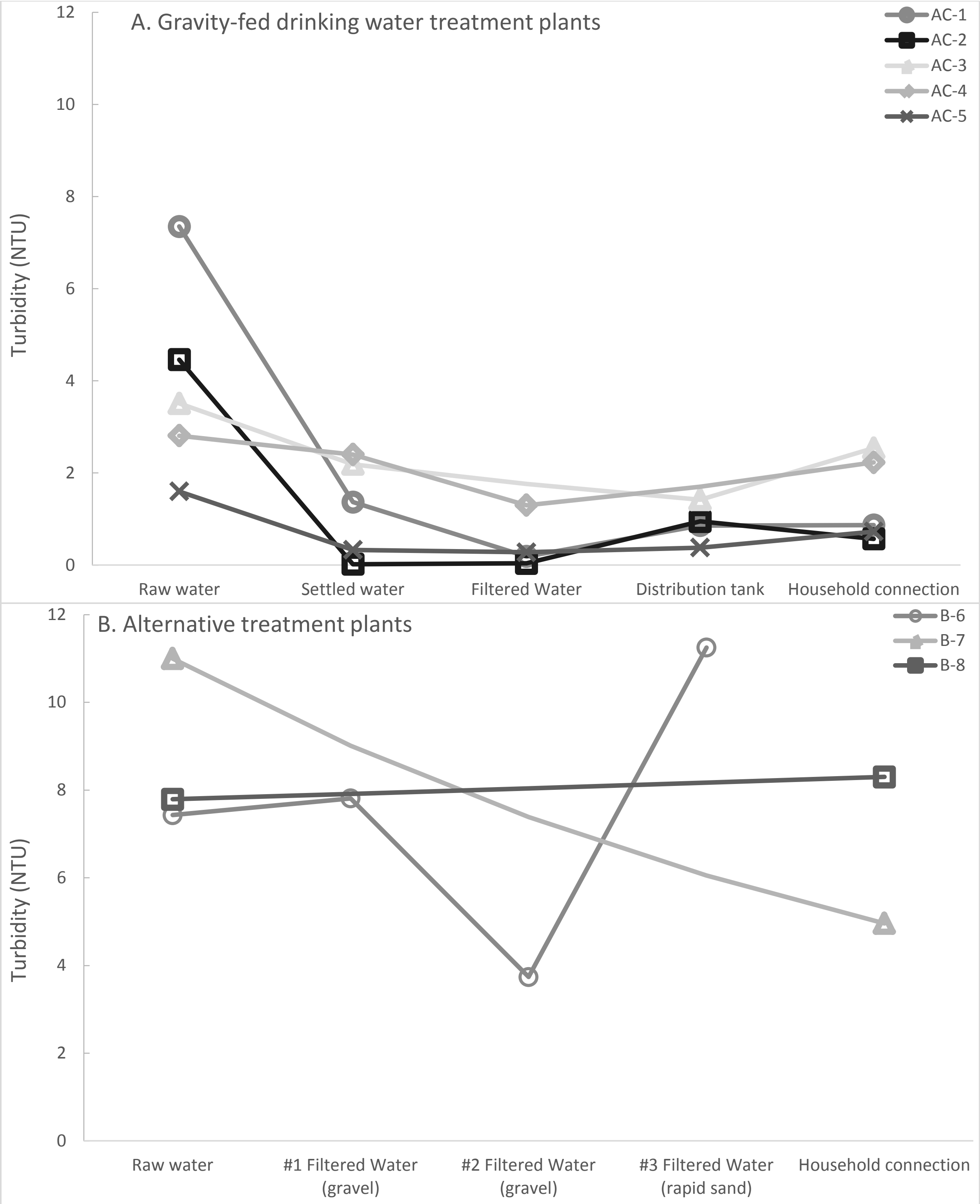

Figure S2: Comparison of turbidity measurements (NTU) from the treatment train processes and at a distant household connection from **(A)** from five gravity-fed water treatment plants (AC-1 through 5)[a];

and **(B)** from three alternative treatments (B6, B-7, and B-8)[b,c] during the dry season. Each data point represents one measurement.

[a]AC-3 did not have a filtration process.

[b]Chlorination was the only treatment for B-7 and B-8.

[c]B-6 included three consecutive filtration processes. Chlorination was not functioning during sampling and we did not collect a sample at a household connection.
